# A novel transcription factor UvCGBP1 regulates development and virulence of rice false smut fungus *Ustilaginoidea virens*

**DOI:** 10.1080/21505594.2021.1936768

**Published:** 2021-08-04

**Authors:** Xiaoyang Chen, Pingping Li, Hao Liu, Xiaolin Chen, Junbin Huang, Chaoxi Luo, Guotian Li, Tom Hsiang, David B. Collinge, Lu Zheng

**Affiliations:** a State Key Laboratory of Agricultural Microbiology/Hubei Key Laboratory of Plant Pathology, Huazhong Agricultural University, Wuhan, Hubei, China; b School of Environmental Sciences, University of Guelph, Guelph, Ontario, Canada; c Department of Plant and Environmental Sciences, Faculty of Science, University of Copenhagen, Frederiksberg C, Denmark

**Keywords:** *Ustilaginoidea virens*, UvCGBP1, MAPK pathway, ChIP-seq, virulence

## Abstract

*Ustilaginoidea virens*, causing rice false smut (RFS) is an economically important ascomycetous fungal pathogen distributed in rice-growing regions worldwide. Here, we identified a novel transcription factor UvCGBP1 (Cutinase G-box binding protein) from this fungus, which is unique to ascomycetes. Deletion of *UvCGBP1* affected development and virulence of *U. virens*. A total of 865 downstream target genes of UvCGBP1 was identified using ChIP-seq and the most significant KEGG enriched functional pathway was the MAPK signaling pathway. Approximately 36% of target genes contain the AGGGG (G-box) motif in their promoter. Among the targets, deletion of *UvCGBP1* affected transcriptional and translational levels of *UvPmk1* and *UvSlt2*, both of which were important in virulence. ChIP-qPCR, yeast one-hybrid and EMSA confirmed that UvCGBP1 can bind the promoter of *UvPmk1* or *UvSlt2*. Overexpression of *UvPmk1* in the *∆UvCGBP1-33* mutant restored partially its virulence and hyphae growth, indicating that UvCGBP1 could function via the MAPK pathway to regulate fungal virulence. Taken together, this study uncovered a novel regulatory mechanism of fungal virulence linking the MAPK pathway mediated by a G-box binding transcription factor, UvCGBP1.

## Introduction

RFS caused by *Ustilaginoidea virens* (teleomorph: *Villosiclava virens*), is a devastating grain disease in main rice planting areas worldwide, especially in China, India and Southeast Asia [1,2]. The occurrence of RFS can cause both serious yield loss and grain contamination by cyclopeptide mycotoxins [3,4]. As a biotrophic phytopathogens, *U. virens* has evolved a specific way to maintain its survival within rice. On the surface of rice spikelets, the conidia of the fungus germinate and the hyphae extend into spikelets' inner space through the small gaps between lemma and palea, thereby colonizing floral organs [5–7]. In floral organs, *U. virens* preferentially attacks the stamen filaments and hijacks rice nutrition to promote the growth of fungus. Thus, this infection style could provide enough nutrition for the formation of rice smut balls [7,8]. Comparative genomics and transcriptomic analyses of *U. virens* infection of rice offer numerous important information for pathogenic mechanisms, of which many pathogenicity factors have been predicted [9]. To date, some pathogenicity genes of *U. virens* have been experimentally confirmed, including *Uvt3277, UvAc1, UvPdeH, SCRE1, SCRE2* (*UV_1261*), *UvBI-1, UvPmk1, UvCDC2, UvCdc3, UvCdc10, UvCdc11, UvCdc12, UvPal1, UvHox2, UvRpd3, UvATG8, UvEC1, UvCom1* and *UvPRO1* [10–23]. An understanding of the pathogenic mechanism of *U. virens* could provide new effective strategies to control RFS.

Transcriptional regulation by a variety of transcription factors is one of the main ways to control cellular development and virulence in plant pathogens. The *U. virens* genome has been predicted to possess over 300 transcription factors, but only three of them (*UvCom1, UvHox2* and *UvPRO1*) have been characterized, and the functions of transcription factors in *U. virens* are largely unknown. DNA motif identification plays a fundamental role in the elucidation of regulatory mechanisms of transcription factors. Among the many reported motifs, the AGGGG motif (G-box) has been observed in the promoters of fungal chitinase, cutinase and many other genes [24–26]. In *Saccharomyces cerevisiae*, the transcriptional activators Msn2p and Msn4p function via upstream stress-response elements (STREs) containing the consensus core G-box sequence, and participate in multi-stress responses [27]. In *Yarrowia lipolytica*, C_2_H_2_-type zinc finger protein MHY1 binds the G-box motif of STREs [28]. In *Trichoderma atroviride*, the C_2_H_2_ zinc-finger protein Seb1 binds the G-box motif for the functions in the response to high osmotic stress [29]. In *Haematonectria haematococca*, it has been proven that the G-box motif in a cutinase gene promoter is required for maintaining the basal level of cutinase gene transcription [26]. Despite this information, mechanistic insights into how the G-box binding transcription factor governs fungal infection remain unexplored.

Here, we identified a novel C_2_H_2_ transcription factor, which was annotated as G-box binding protein, and the disruption of *UvCGBP1* affected the development and virulence of *U. virens*. We used ChIP-seq to identify over 800 target genes and to provide a comprehensive genome-wide binding profile of UvCGBP1 and its regulatory network. Functional analysis indicated that the UvCGBP1-mediated MAPK pathway regulated the virulence of *U. virens*. Our results provide new insights into the mechanism of UvCGBP1-mediated gene regulation in *U. virens*.

## Materials and methods

### Fungal strains, culture and transformation

The reported *U. virens* strain HWD-2 was used as the wild-type (WT) strain [30]. The WT and all transformed strains (Table 1) were cultured on potato sucrose agar (PSA) at 28°C. The conidia collected from the potato sucrose broth (PSB) medium were used for *Agrobacterium tumefaciens* mediated transformation (ATMT) based on the method of Chen et al. [18].

**Table 1.** Wild-type and mutant strains of *U. virens* used in this study

Strain	Description	Reference
WT	Wild-type strain, HWD-2	Jia et al., 2015
Δ*UvCGBP1-33*	*UvCGBP1* deletion mutant of HWD-2	This study
Δ*UvCGBP1-36*	*UvCGBP1* deletion mutant of HWD-2	This study
CΔ*UvCGBP1-33*	*UvCGBP1* complementation strain from Δ*UvCGBP1-33*	This study
UvCGBP1-3	GFP-UvCGBP1 protein subcellular localization strain	This study
∆*Uv8b_2652*	*Uv8b_2652* deletion mutant of HWD-2	This study
Δ*Uv8b_7184*	*Uv8b_7184* deletion mutant of HWD-2	This study
Δ*Uv8b_7527*	*Uv8b_7527* deletion mutant of HWD-2	This study
Δ*Uv8b_8131*	*Uv8b_8131* deletion mutant of HWD-2	This study
Δ*UvPmk1-28*	*UvPmk1* deletion mutant of HWD-2	Tang et al., 2019
Δ*UvSlt2-2*	*UvSlt2* deletion mutant of HWD-2	This study
*OEUvPmk1-1* *OEUvPmk1-2*	*UvPmk1* overexpression in Δ*UvCGBP1-33* mutant *UvPmk1* overexpression in Δ*UvCGBP1-33* mutant	This study This study
*OEUvSlt2-1* *OEUvSlt2-2*	*UvSlt2* overexpression in Δ*UvCGBP1-33* mutant *UvSlt2* overexpression in Δ*UvCGBP1-33 *mutant	This study This study

To measure the vegetative growth, 5-mm-diameter mycelial plugs were inoculated onto fresh PSA plates, incubated at 28°C for 14 days and the colony diameter was measured. To measure the condition and dry hyphal weight, the strains were cultured in PSB shaken at 180 rpm at 28°C for 7 days. The cultures were filtered through four layers of gauze to collect spores, and conidiation was determined using a hemocytometer. Mycelia were collected, dried and weighed. For stress response tests, 5-mm-diameter mycelial plugs were cultured on PSA alone or PSA containing exogenous 0.03% Sodium dodecylsulphate (SDS) (w/v), 0.5 M Sorbitol, 0.12 mg/ml Congo red (CR), 0.3 M NaCl, 0.12 mg/ml Calcofluor white (CFW) or 0.015% H_2_O_2_. The colony diameters were measured after being incubated for 14 days and the inhibition rates of various stresses were calculated. Each experiment was repeated three times.

### Molecular phylogenetic analysis

The full sequences of *UvCGBP1*, cutinase genes (*Uv8b_2652, Uv8b_7184, Uv8b_7527* and *Uv8b_8131*) and other *UvCGBP1* target gene sequences were downloaded from the National Center for Biotechnology Information (NCBI). Protein domains were predicted using the software SMART (http://smart.embl-heidelberg.de/). Phylogenetic tree was constructed by the sorftware MEGA 7.0 and the Neighbor-joining (NJ) algorithm involving 1000 bootstrap replicates was used.

### Gene deletion and complementation

To generate *UvCGBP1* knockout mutants, 1.0 ~ 1.5-kb of upstream and downstream flanking sequences of *UvCGBP1* were cloned into pGKO to construct the knockout vector pGKO-UvCGBP1. For complementation experiments, an approximately 4-kb fragment (containing 2-kb native promoter region and *UvCGBP1* gene without termination codon) was ligated with the pNeo3300III vector. The EHA105 strain with the pGKO-UvCGBP1 and pNeo3300III-UvCGBP1 vectors were transformed with ATMT method by co-culture with conidia of the WT or Δ*UvCGBP1-33*, respectively. Transformed strains were confirmed by RT-PCR and Southern blot analysis. Based on the protocol of Amersham Gene Images Alkphos Direct Labeling and Detection System (GE Healthcare, UK), southern blot analysis was conducted. Gene deletion and complementation of other UvCGBP1 target genes were performed using similar methods described above.

### Pathogenicity and rice infection assays

The inoculation method of *U. virens* strains follows Jia et al. [30]. The susceptible rice cv. Wanxian-98 was used in all pathogenicity assays. At the early booting stage, the middle section of distal internodes of rice plants was inoculated with 2 ml of mycelial/conidial suspensions (1×10^6^ conidia ml^−1^) using a syringe. After 21 days post-inoculation (dpi), the average number of rice smut balls per panicle was calculated for statistical analysis. For infection assays, inoculated rice spikelets at 1 and 6 dpi were collected, treated in 2.5% glutaraldehyde fixative, and then used for infection observation by scanning electron microscopy (SEM). Each experiment was repeated three times.

### Cutinase activity assay

The cutinase activity was measured based on the method of Wang et al. [31]. The ρ-nitrophenyl butyrate (ρ-NPB) was used as chromogenic substrate. Each reaction mixture consisted of 200 μl of crude enzyme solution, 100 μl of 0.8% Triton X-100, 690 μl of 0.1 M sodium phosphate buffer (pH = 7.0) and 20 μl of 1.76% (v/v) ρ-nitrophenyl butyrate in acetonitrile. The reactions were conducted at 37°C for 10 min. The absorbance (OD value) was measured with a spectrophotometer (Mapuda, China) at 405 nm wavelength to calculate the cutinase activity. All determinations were repeated ten times.

### Generation of GFP-UvCGB1 fusion construct

The full-length *UvCGBP1* coding region was cloned into pNeo3300III-GFP vector, and the constructed pNeo3300III-GFP-UvCGBP1 vector was transformed into *A. tumefaciens* EHA105 cells. The EHA105 strain was co-cultured with the conidia of the ∆*UvCGBP1-33* using ATMT method. Transformants were verified with PCR and western blotting. The GFP-tagged strain UvCGBP1-3 was chosen for subcellular localization observation by confocal microscopy.

### RNA-seq analysis

Seven-day-old mycelia of the WT and Δ*UvCGBP1-33* strains were collected from PSB culture and used for total RNA isolation. RNA-seq libraries were created using an Illumina TruSeq RNA Sample Preparation Kit and then sequenced on an Illumina HiSeq 4000 system (IGENEBOOK Biotechnology Co., Ltd, Wuhan, China) based on HiSeq 4000 SBS Kit (300 cycles) protocol. Three biological replicates were carried out for each strain. The raw data of paired-end reads were filtered by SeqPrep and Sickle with default parameters. The software Tophat (version 2.0.14) and Cufflinks (version 2.2.1) were used to map clean reads to the *U. virens* genome and to calculate differential expression [32]. Filter conditions fold change >2 and adjusted *p* < 0.05 were applied for identification of differentially expressed genes [33–35].

### Chromatin Immunoprecipitation and ChIP-seq data analysis

Seven-day-old mycelia of the GFP-tagged strain UvCGBP1-3 collected from PSB was used for Chromatin immunoprecipitation (ChIP), and ChIP was performed with anti-GFP tag antibody-ChIP grade (Abcam, MA). Libraries were constructed with the DNA samples of *UvCGBP1-3* based on the protocol of the Illumina TruSeqCHIP Sample Prep Set A. Libraries were sequenced on an Illumina Hiseq2000 with PE150 by Wuhan IGENEBOOK Biotechnology Co., Ltd. For ChIP-seq data analysis, genomic repeats were filtered out, and the unique reads were mapped to the *U. virens* genome. Peaks of UvCGBP1 were found with MACS software. The signal density was normalized and calculated within a window 4-kb upstream to 4-kb downstream of TSSs of coding and non-coding targets. The enriched motifs of UvCGBP1 were generated by HOMER. All peak signals were viewed with the Integrative Genomics Viewer (IGV, version 2.7.2). The *P* values in ChIP-seq data were calculated with default parameters [36–39].

### RNA manipulation, qRT-PCR and ChIP-PCR analysis

Total RNAs of the mycelia and infected rice spikelets (1, 3, 6, 9, 13 and 20 dpi) were extracted using an RNA Kit 200 (OMEGA). cDNA templates were prepared with the kit TransScript® One-Step gDNA Removal and cDNA Synthesis SuperMix. The TransStart® Tip Green qPCR SuperMix (TransGen Biotech, China) was used to perform quantitative real-time PCR (qRT-PCR), and qRT-PCR assays ran on a CFX96^TM^ real-time PCR system. Levels of the *U. virens* β-tubulin gene (*Uv8b_900*) and Input were used for normalization in qRT-PCR and ChIP-PCR, respectively. The data were taken from three biological replicates.

### Transactivation activity assays

The transactivational activity assays were carried out using Matchmaker pGBKT7 (Clontech, USA). The cDNA sequence of *UvCGBP1* was inserted into pGBKT7, and the pGBKT7-UvCGBP1 vector was transformed into Y2HGold cells. The transformants were cultured with SD/-Trp medium (Clontech) and confirmed by PCR. Transactivation activity of transformed strains was tested on SD/-Trp-His medium (Clontech) using suitable X-α-Gal.

### Yeast one-hybrid assay

The *UvCGBP1* cDNA sequence was ligated to pGADT7 as the prey, and approximately 50-bp sequence of the promoter region of the UvCGBP1 target gene from ChIP was ligated to the pAbAi vector (Clontech) as a bait. The pAbAi::pro vectors were transformed into Y1HGold (Clontech) cells, and transformed strains were isolated with SD/-Ura medium and confirmed by PCR. Then, the pGADT7-UvCGBP1 vector was transformed into Y1HGold cells harboring the pAbAi::pro vector, and the transformants were isolated with SD/-Ura media with 500 ng/ml Aureobasidin A (AbA). The p53-AbAi vector transformants were used as the negative control and pAbAi::pro vector transformants as the positive control.

### Purification of UvCGBP1 protein and EMSA

The cDNA sequence of UvCGBP1 was ligated to the pGEX-4T-2 vector, and the pGEX-4T-2-UvCGBP1 vector was then transformed into BL21 (DE3) strain. The expression of fusion protein UvCGBP1-GST was induced as described in Molecular Cloning: A Laboratory Manual (III). Briefly, this strain was cultured in 50 ml ampicillin-resistance LB medium in shake flasks (180 rpm) at 37°C for 12 h. Then, the cells were induced with 0.1 M IPTG at 28°C for 8 h and harvested. The cells were washed with PBS buffer and disrupted with ultrasound to extract protein. The UvRpd3-GST protein was purified with GSTrap^TM^ (GE Healthcare, USA). A total of 1 ml GSTrap was added to the chromatography column, and washed three times with 500 µl PBS. The extracted protein solution was passed through the column, washed three times with PBS, and finally eluted with the eluent buffer (50 mM Tris-HCl, 10 mM GST). Electromobility shift assays (EMSA) were conducted using a LightShift Chemiluminescent EMSA kit (Thermo, Rockford).

### Western blot analysis

For western blot analysis, the proteins separated on SDS-PAGE gels were transferred onto a polyvinylidene fluoride membrane with a BioRad electroblotting apparatus as described [40]. The signals on the blots were detected with anti-p44/42 MAP kinase antibody (Cell Signaling Technology, MA) or anti-GFP antibody (Thermo Fisher Scientific, USA) under the ECL Supersignal system (Pierce, Rockford, IL).

### Light, fluorescence and scanning electron microscopy

Fungal morphology of *U. virens* strains was observed by a light microscopy (Carl Zeiss, Jena, Germany). Subcellular localization of GFP-UvCGBP1 was photoed using a Zeiss LSM 510 Meta confocal fluorescence microscope, with the combinations of excitation and emission wavelengths of 488 nm/bandpass 500 to 550 nm for GFP. The infection process of *U. virens* on rice spikelets was viewed using a JEOL JSM-6390LV SEM.

### Statistical analyses

Data were subjected to analysis of variance (ANOVA) and means were separated by the Least Significant Difference (LSD) test (*P* = 0.05).

### Availability of data and materials

ChIP-seq data generated in this study was deposited in the NCBI Gene Expression Omnibus (GEO) database with accession number GSE141697. RNA-seq data were deposited in the NCBI Sequence Read Archive (SRA) database with accession number SRR14292001.

## Results

### Identification of UvCGBP1 in *U. virens*

**Figure 1.**
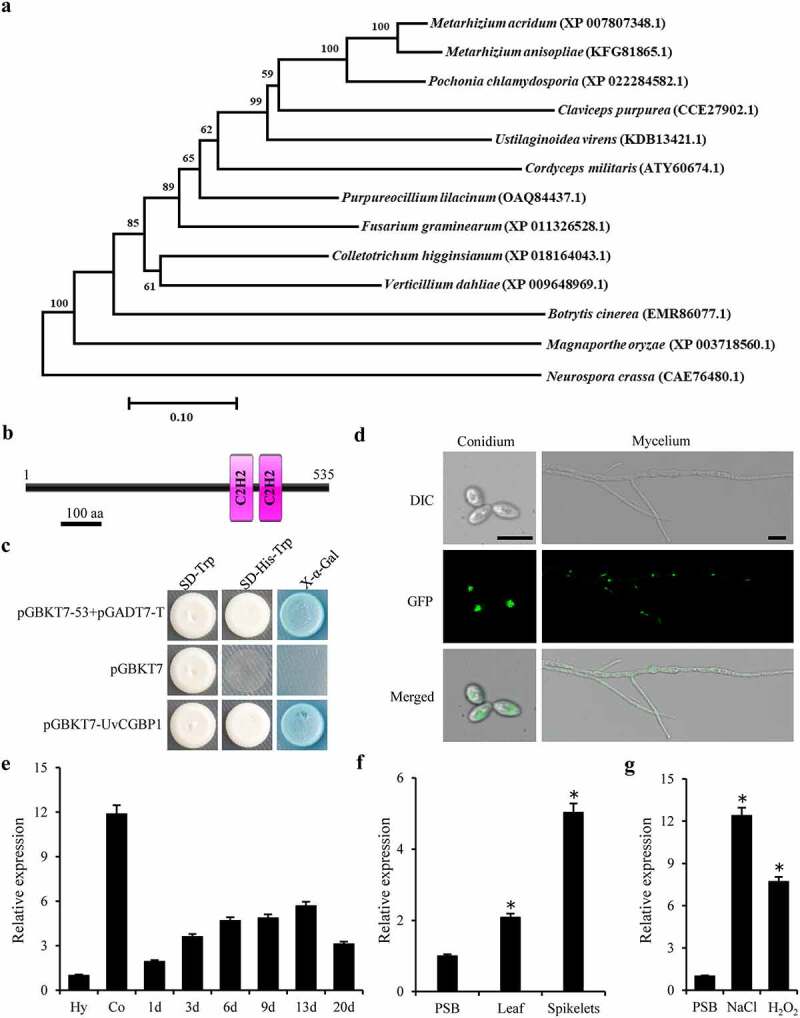
Identification of UvCGBP1 in *U. virens*. (a) NJ tree of putative CGBP1 homologues from 13 fungal genomes generated with MEGA7.0. The bootstrap percentage values from 1000 repeats are shown at branch nodes. (b) Predicted Pfam domain of C_2_H_2_ zinc finger (box) protein UvCGBP1. (c) Transactivation analysis of UvCGBP1 in yeast. The vectors pGBKT7-53/pGADT7-T and pGBKT7 were expressed in yeast as positive and negative controls, respectively. (d) Subcellular localization of UvCGBP1 in *U. virens*. DIC, differential interference contrast; GFP, green fluorescent protein. Scale bar = 10 μm. (e) qRT-PCR detected expression of *UvCGBP1* in conidia (Co), hyphae (Hy) and different infection stages on rice spikelets (1–20 d). (f) Expression analysis of *UvCGBP1* after treated with rice leaf and spikelet extracts with qRT-PCR. (g) Expression analysis of *UvCGBP1* after treated with chemical, 1 M NaCl or 10 mM H_2_O_2_ with qRT-PCR. Asterisks represent significant differences between PSB control and stress treatments based on LSD at *P* = 0.05

In our transcriptome data of *U. virens* infecting rice [41], we found that *UvCGBP1* was significantly up-regulated (Figure S1(a)). By using Blastp in NCBI NR database, CGBP1 homologues were only found in ascomycetes but not in prokaryotes and other eukaryotes, such as animals and plants, and other fungi such as basidiomycetes. Phylogenetic tree was generated with CGBP1 homologues from 13 ascomycete fungi, and the results revealed that UvCGBP1 was most similar to CGBP1 protein of *Pochonia chlamydosporia* (Figure 1(a)). Functional domain analysis demonstrated that *UvCGBP1* contained two conserved C_2_H_2_-zinc finger domains (Figure 1(b)). To further examine transcriptional activity of *UvCGBP1*, yeast one-hybrid assay was used to assess transcriptional activation of *UvCGBP1*, and this gene showed trans-activation activity in yeast (Figure 1(c)). These suggest that *UvCGBP1* acts as a C_2_H_2_-type zinc finger transcription factor and is specifically distributed in ascomycetes.

### Subcellular localization and expression patterns of UvCGBP1 in *U. virens*

To confirm the subcellular localization of the transcription factor UvCGBP1, the pNeo3300III-GFP-UvCGBP1 vector was transformed into the ∆*UvCGBP1*-33 Transformed strains were verified by PCR and western blotting (Figure S1(b)). Under confocal microscopy, the GFP signal in the UvCGBP1-3 strain was observed in the nucleus of both conidia and vegetative hyphae (Figure 1(d); S1(c)), indicating that GFP-UvCGBP1 was localized in the nucleus of *U. virens*.

The expression profile of *UvCGBP1* in different stages of mycelium growth, conidiation and infection processes was determined by qRT-PCR. *UvCGBP1* expression was significantly up-regulated during conidiation and infection processes (Figure 1(e)). The result suggested that *UvCGBP1* might be involved in conidial production and spikelet colonization of *U. virens*.

### UvCGBP1 was highly expressed after induction by rice extract and abiotic stress

To investigate the upstream activator of *UvCGBP1*, we used several abiotic stresses and rice spikelet/leaf extract for induction treatments. The expression profiles of *UvCGBP1* under different abiotic stresses and rice spikelets/leaf extract treatments were measured, and the transcript level of *UvCGBP1* was strongly induced by rice extract, especially by spikelets extract (Figure 1(f)). Moreover, the expression of *UvCGBP1* was up-regulated under NaCl osmotic stress and oxidative stress H_2_O_2_ (Figure 1(g)). These results suggested that *UvCGBP1* could be activated by some abiotic stresses and exposure to rice extracts.

### Deletion of UvCGBP1 affected mycelial growth, morphogenesis, conidiation and various morphogenesis- and pathogenesis-associated stress response

**Figure 2.**
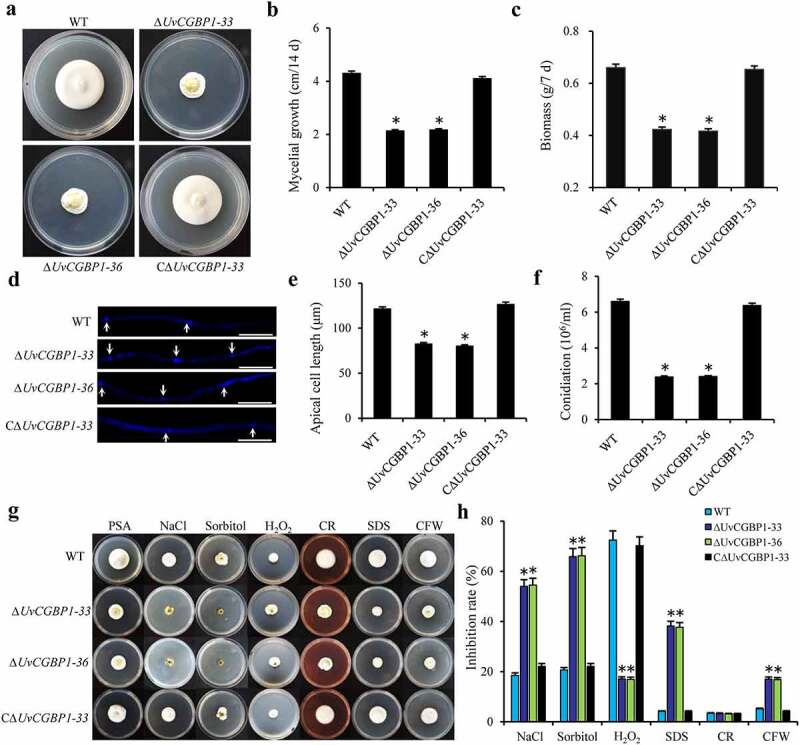
Deletion of *UvCGBP1* affects hyphal growth, conidiation and various stress response. (a) Colony morphology of the WT, Δ*UvCGBP1-33*, Δ*UvCGBP1-36* and CΔ*UvCGBP1-33* strains on PSA for 14 days. (b) Colony diameter of the mutant strains on PSA for 14 days. (c) Mycelial biomass of the mutant strains. (d) Hyphal tips stained by CFW. Scale bar = 60 μm. (e) The apical compartment length of the mutant strains. (f) Conidial production of the mutant strains from PSB at 180 rpm for 7 days. (g) Colony growth of the mutant strains on PSA supplied with 0.3 M NaCl, 0.5 M sorbitol, 0.015% H_2_O_2_, 0.03% SDS, 0.12 mg/ml CR or 0.12 mg/ml CFW after incubation of 14 days at 28°C. (h) Inhibition rate of colony growth by various stress inducers. Asterisks represent significant differences between the WT and mutants based on LSD at *P* = 0.05

To further investigate the functions of *UvCGBP1*, the *UvCGBP1* gene was deleted using a homologous recombination strategy (Figure S2(a)). The knockout mutants ∆*UvCGBP1-33* and ∆*UvCGBP-36*, and complementation strain CΔ*UvCGBP1-33* confirmed by PCR, RT-PCR or southern blot analyses (Figure S2(b-d); Table S1). RT-PCR analyses demonstrated that the *UvCGBP1* gene was not expressed in either ∆*UvCGBP1-33* or ∆*UvCGBP-36*, but it was expressed in the WT and complementation strain CΔ*UvCGBP1-33* (Figure S2(c)). Southern blotting showed that a 6.2-kb band was detected in the mutants ∆*UvCGBP1-33* and ∆*UvCGBP-36*, in contrast with a 3.2-kb band in the WT. A 3.2-kb band and a band of unknown size were found in the complementation strain CΔ*UvCGBP1-33* (Figure S2(d)). Hence, the deletion mutants ∆*UvCGBP1-33* and ∆*UvCGBP-36*, and complementation strain CΔ*UvCGBP1-33* were confirmed and further chosen for functional characterization of *UvCGBP1*. After being cultured on PSA or PSB medium, vegetative growth rates and mycelial dry weights of ∆*UvCGBP1* mutants were both significantly reduced when compared with those of the WT and CΔ*UvCGBP1-33* strains (Figure 2(a,b,c)). Length of hyphal tip cells of the ∆*UvCGBP1* mutants was significantly reduced (Figure 2(d,e)). Deletion of *UvCGBP1* caused significantly decreased conidial production (Figure 2(f)). To explore the roles of *UvCGBP1* in the regulation of different stress responses, mutant strains were cultured in PSA containing CFW, NaCl, sorbitol, H_2_O_2_, SDS or CR (Figure 2(g)). The results indicated that *∆UvCGBP1* mutants were more sensitive to the chemical treatment of NaCl, sorbitol, SDS or CFW but exhibited an increased resistance to H_2_O_2_ when compared with the WT and CΔ*UvCGBP1-33* strains. In the presence of CR in PSA, similar growth rates were found between the WT and *∆UvCGBP1* strains (Figure 2(h)). These indicate that *UvCGBP1* is involved in response to osmotic, oxidative and cell wall integrity stresses.

Overall, the results suggest that *UvCGBP1* is necessary for mycelial growth, cell morphogenesis, conidiation and various morphogenesis- and pathogenesis-associated stresses of *U. virens.*

### UvCGBP1 was essential for spikelet colonization and virulence of U. virens

**Figure 3.**
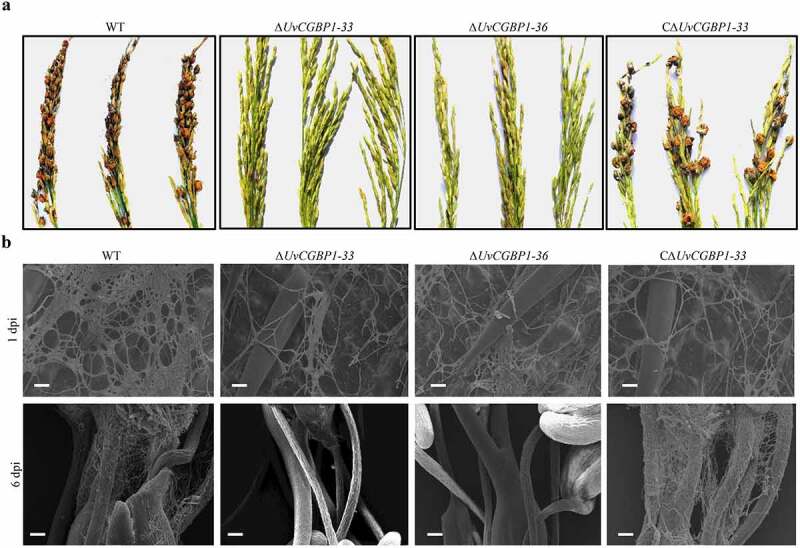
*UvCGBP1* is important for invasive growth and virulence. (a) Virulence assays of the WT, Δ*UvCGBP1-33*, Δ*UvCGBP1-36* and CΔ*UvCGBP1-33* strains on rice spikelets at 21 dpi. (b) Infection observation in inoculated rice spikelets at 1 and 6 dpi by SEM. Scale bars = 20 μm (1 dpi) and 100 μm (6 dpi)

To explore the role of *UvCGBP1* in *U. virens* infection, we first conducted pathogenicity tests for the WT and *UvCGBP1* mutant strains on susceptible rice cv. Wanxian-98 at the early booting stage. After 21 days post-inoculation (dpi), the WT produced approximately 50 false smut balls on spikelets per panicle while CΔ*UvCGBP1-33* produced approximately 35 false smut balls on spikelets per panicle. In contrast, the *∆UvCGBP1* mutants did not produce any rice smut balls on rice spikelets, and completely lost pathogenicity (Figure 3(a)).

In plant infection assays, for all the tested strains, numerous hyphae were observed to spread on the surface of rice spikelets at 1 dpi. At 6 dpi, hyphae of the WT or CΔ*UvCGBP1-33* were found in the inner space of rice spikelets and filaments were fully infected. In contrast, the *∆UvCGBP1* mutants did not extend into the spikelets and failed to produce observable hyphae on the surface of filaments (Figure 3(b)). These results revealed that *UvCGBP1* was important for virulence of *U. virens.*

### UvCGBP1 regulating virulence might be independent of cutinases

**Figure 4.**
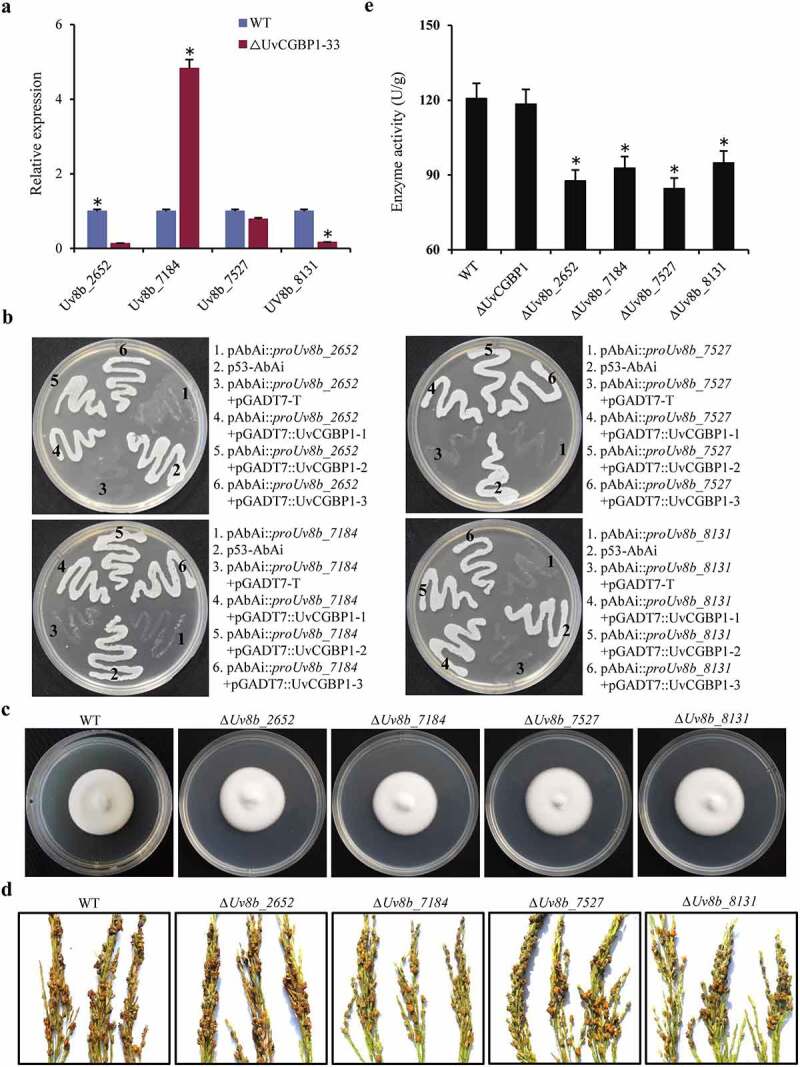
UvCGBP1 regulating virulence might be independent of cutinase genes. (a) Expression of cutinase genes in the WT and *∆UvCGBP1-33* by qRT-PCR relative to β-tubulin. (b) Yeast one-hybrid analysis indicated *UvCGBP1* can bind to the promoter of these four cutinase genes (*Uv8b_2652, Uv8b_7184, Uv8b_7527* and *Uv8b_8131*). (c) Colony morphology of the WT and cutinase gene deletion mutants on PSA for 14 days. (d) Virulence assays of the WT and cutinase gene deletion mutants on rice spikelets at 21 dpi. (e) Determination of cutinase activity in 7-day-old hyphae from PSB between the *∆UvCGBP1-33*, cutinase gene deletion mutants and WT. Asterisks represent significant differences between WT and mutants based on LSD at *P* = 0.05

Most fungal pathogens produce cutinases that can hydrolyze host cutin and promote pathogen invasion. Since UvCGBP1 was annotated as a putative cutinase G-box binding protein [9], we attempted to explore whether the loss of pathogenicity of *∆UvCGBP1* mutant was related to the deletion of cutinase genes and to verify the functional link between UvCGBP1 and cutinases. In *U. virens*, four cutinase genes (*Uv8b_2652, Uv8b_7184, Uv8b_7527* and *Uv8b_8131*) were found in the genome. In the *∆UvCGBP1-33*, the expressions of *Uv8b_2652* and *Uv8b_8131* were significantly downregulated, whereas *Uv8b_7184* was significantly upregulated in comparison with the WT (Figure 4(a)). Yeast one-hybrid results showed that *UvCGBP1* can bind the G-box region to promoters of the four cutinase genes (Figure 4(b)). To investigate the role of cutinase in pathogenicity, the four cutinase genes were individually deleted. However, the deletion of each cutinase gene separately did not cause abnormal mycelial growth or pathogenicity (Figure 4(c,d)). We next assessed the cutinase activity, and found in *∆UvCGBP1-33* that it was not changed in hyphae but significantly reduced for each of the four cutinase gene deletion mutants (Figure 4(e)). Taken together, these results indicate regulation of virulence by UvCGBP1 might be independent of targeted cutinase genes in *U. virens*, suggesting that UvCGBP1 could modulate virulence via regulating other targets in *U. virens*.

### Genome-wide identification of target genes bound by UvCGBP1

**Figure 5.**
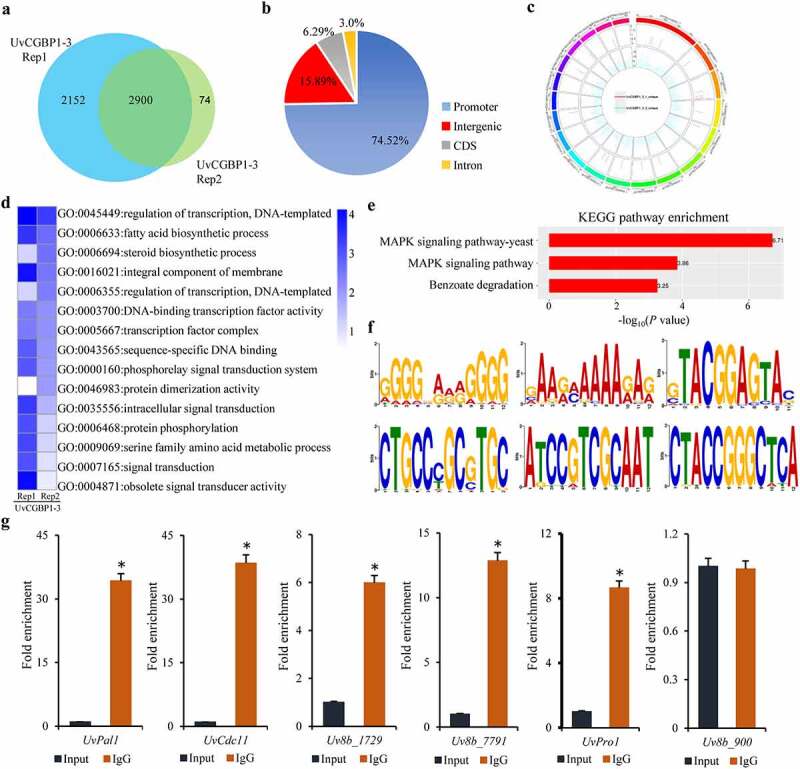
Genome-wide binding sites of UvCGBP1 identified by ChIP-Seq. (a) UvCGBP1 binding peaks and target genes identified by ChIP-Seq. (b) Distribution of UvCGBP1-binding sites. (c) UvCGBP1-binding sites were shown in the genome of *U. virens*. (d) Functional categories of UvCGBP1-bound genes. (e) KEGG enrichment analysis of UvCGBP1-bound gene-associated ChIP-seq data set. (f) Identification of UvCGBP1 binding motif with MEME (Top six). (g) ChIP-qPCR validated ChIP-seq results using five selected targets. Non-target gene, *Uv8b_900* (β-tubulin), was used as a negative control. Asterisk means that UvCGBP1 was significantly enriched on the promoter of tested target gene (*P* = 0.05)

To identify global targets of the transcription factor UvCGBP1 in *U. virens*, we used the GFP-UvCGBP1 target strain *UvCGBP1-3* to perform ChIP-seq analysis. Using western blot analysis, the level of GFP-UvCGBP1 fusion protein was confirmed (Figure S1(b)). These samples were deep-sequenced to derive approximately 26–39 million reads in the ChIP-Seq data, which were mapped with the *U. virens* genome using the ultrafast Bowtie aligner (Table S2). Approximately 98% of the enriched peaks of the first experiment were found in the second experiment. A total of 5126 peaks was identified, and 2900 peaks were common to the two independent experiments (Figure 5(a)). Among these, 74.52% of the peaks were located in promoter regions, 15.89% of the peaks in the intergenic regions, 6.29% of the peaks in coding sequence (CDS) and 3% of the peaks in introns (Figure 5(b)). A histogram showed the length of contigs of genomic sequence reads that are UvCGBP1-bound indicated that the majority of binding sites encompassed 100–1000 bp of chromosomal sequences (Figure S3(a)). The data set of UvCGBP1-bound gene-associated sites was examined for the position of the binding site relative to the transcription start site (TSS), and the results showed that binding sites could lie up to 4 kb upstream of the TSS (Figure S3(b)). Based on the false discovery rate (FDR) <0.05 and fold enrichment over input >2, the numbers of target genes predicted in the two experiments were 865 genes common to both. This genome-wide overview revealed that enriched regions were distributed throughout the *U. virens* genome (Figure 5(c)). The data set of the 865 UvCGBP1-bound genes were examined for their functional categories to identify pathways downstream of UvCGBP1-bound genes, and these gene classes were found to be enriched for various functional categories, such as transcription factor complex (GO:0005667), regulation of transcription (GO:0045449), signal transduction (GO:0007165), and protein phosphorylation (GO:0006468) (Figure 5(d)). KEGG enrichment analysis showed the most significantly enriched functional pathway was the MAPK signaling pathway and benzoate degradation (Figure 5(e)). By multiple EM for motif elicitation (MEME) of the identified peaks, an UvCGBP1-bound DNA motif was discovered, and a C/AAGGGG motif, a G/AAAAAA motif, a CTGCCCGCTGC motif, a ATCCGTCGCAAT motif, a CTACCGGGCTCA motif and a TACGGAGTA motif were found to comprise most of UvCGBP1-bound DNA motifs (Figure 5(f)). To validate the UvCGBP1 binding targets identified in the ChIP-seq experiments, we performed ChIP-qPCR using primers specific to the promoters of selected targets*Uv8b*_*1729, Uv8b_7791, UvPro1, UvPal1* and *UvCdc11*. Compared with the negative control β-tubulin gene (*Uv8b*_*900*), the promoter regions of these five genes were enriched (Figure 5(g)), confirming UvCGBP1-specific enrichment of all tested targets.

### RNA-seq analysis of UvCGBP1 confirms UvCGBP1 target genes and associated biological functions

RNA-seq was conducted to compare the global gene expression patterns between the WT and *∆UvCGBP1-33* (Table S3). On the basis of the log_2_ ratio (*∆UvCGBP1-33* mutant/WT) values with a more than or equal to two-fold change, 580 genes were upregulated and 494 genes were downregulated in the *∆UvCGBP1-33* mutant (Figure S3(c,d)). By Gene Ontology (GO) classification, all differentially expressed genes were classified into 32 functional groups and most of them were grouped into metabolic process, binding and catalytic activity (Figure S3(e)). The qRT-PCR results of 10 randomly selected genes revealed that the expression patterns of each upregulated or downregulated gene were consistent with that in the RNA-seq data (Figure S3(f)).

**Figure 6.**
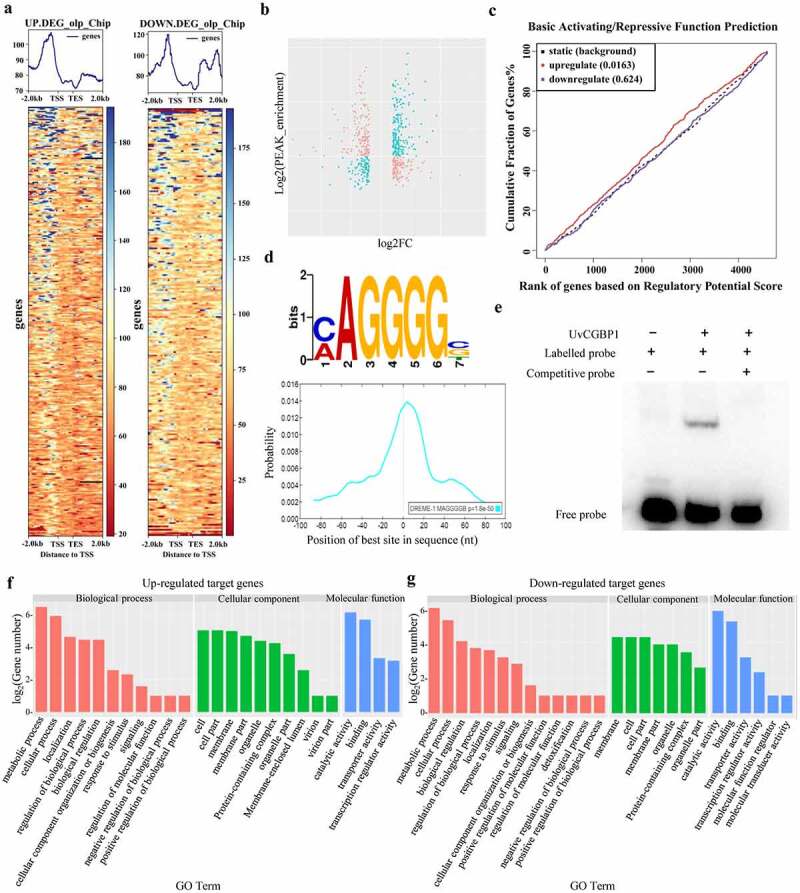
Integrative analysis of RNA-seq and ChIP-seq data. (a) Heatmap and (b) scatter plot indicated up- and down- regulated UvCGBP1-binding target genes. (c) Basic activation/repressive function prediction of UvCGBP1 by BETA software. (d) UvCGBP1-binding target genes significantly enriched in C/AAGGGG motif by MEM-ChIP. (e) EMSA confirmed that UvCGBP1 can bind C/AAGGGG motif. (f,g) Functional categorization of up- and down-regulated UvCGBP1-binding target genes

Joint analysis of ChIP-seq and RNA-seq data was conducted to define regulation patterns of UvCGBP1. By comparing the differentially expressed genes of RNA-seq with the peak genes of ChIP-seq, the genes for differential expression of target genes in ChIP-seq were explored, and 321 genes were found to be upregulated while 255 were downregulated among the 865 target genes of UvCGBP1 (Figure 6(a,b)). Based on basic activation/repressive function prediction, statistical test results indicated that UvCGBP1 was both a transcription activator and transcription repressor (Figure 6(c)). In these upregulated and downregulated target genes, approximately 36% of these genes contained an AGGGG motif in their promoter (Figure 6(d)). Electrophoretic mobility shift assay (EMSA) results revealed that UvCGBP1 can bind to this motif (Figure 6(e)). Functional categories of these upregulated (Figure 6(f)) and downregulated (Figure 6(g)) target genes were found for the categories of metabolism process, cell process, and catalytic activity.

### UvCGBP1 is involved in the regulation of multiple MAPK pathways

**Figure 7.**
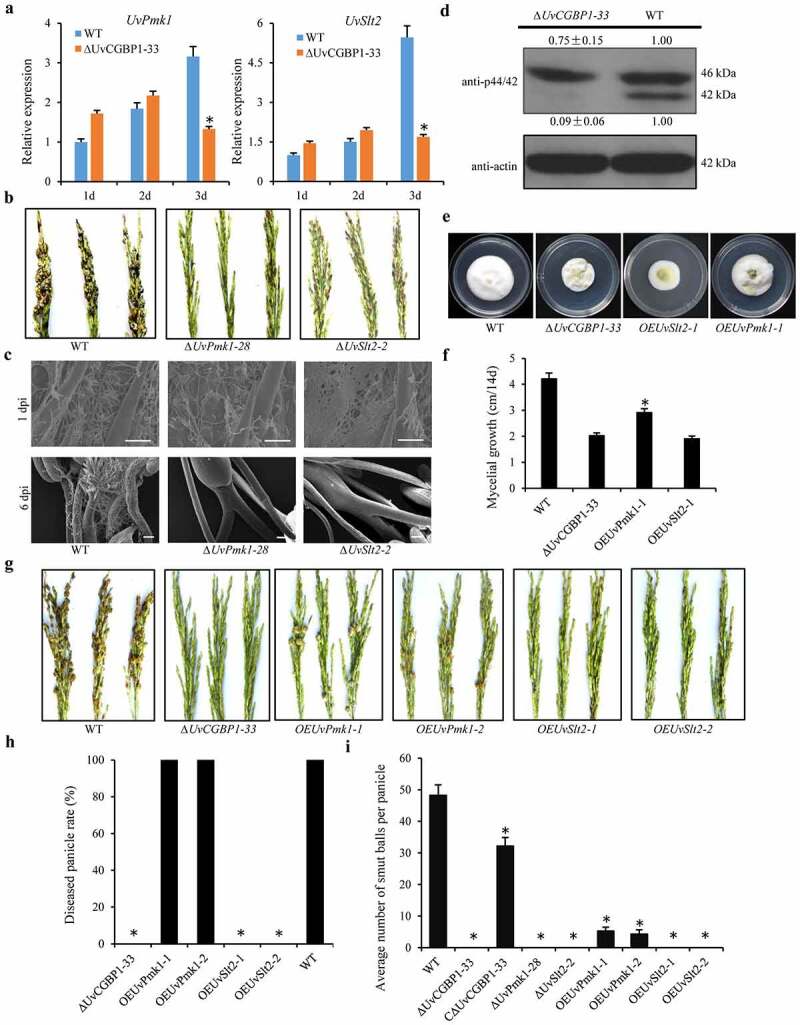
UvCGBP1 regulates virulence through mediating MAPK pathway. (a) Expressions of *UvPmk1* and *UvSlt2* in the WT and *∆UvCGBP1-33* during the early infection process (1–3 dpi) by qRT-PCR. Asterisks represent significant differences between the WT and mutant based on LSD at *P* = 0.05. (b) Virulence assays of *UvPmk1* and *UvSlt2* deletion mutants on rice spikelets at 21 dpi. (c) Infection development of *UvPmk1* and *UvSlt2* deletion mutants on rice spikelets under SEM at 1 and 6 dpi. Scale bars = 50 μm (1 dpi) and 100 μm (6 dpi). (d) Western blot analysis of proteins extracted from PSB-cultured hyphae of the *∆UvCGBP1-33* and WT using anti-p42/44 antibody. The WT loading set was considered as 1.00, and relative quantified signals of each band were calculated. (e) Colony morphology of *UvPmk1* and *UvSlt2* over-expression mutants in *∆UvCGBP1-33* and WT on PSA for 14 days. (f) Mycelial growth of ∆*UvCGBP1-33, OEUvPmk1-1, OEUvSlt2-1* and the WT on PSA for 14 days. Asterisks represent significant differences between the ∆*UvCGBP1-33* and *OEUvPmk1-1* based on LSD at *P* = 0.05. (g) Virulence assay of the over-expression mutants in *∆UvCGBP1-33* on rice spikelets at 21 dpi. (h) Disease incidence in rice panicles. (i) Mean number of smut balls per panicle. Asterisks represent significant differences between the WT and other strains based on LSD at *P* = 0.05

In KEGG enrichment analysis, 35 target genes of UvCGBP1 were closely linked to the MAPK pathway (Table S4). The two key MAP kinases, *UvPmk1* and *UvSlt2*, among the target genes of UvCGBP1, were chosen for target regulation analysis. In the genome browser, ChIP-seq peaks for *UvPmk1* and *UvSlt2* were among the highest ChIP-seq peaks and had relatively high enrichment vs. input control (Figure S4(a)). ChIP-qPCR with primers specific to the promoters of *UvPmk1* and *UvSlt2* confirmed that these genes were enriched effectively, and qRT-PCR assays showed significantly lower transcriptional levels for these two genes in the ∆*UvCGBP1* mutant compared to those of the WT (Figure S4(b,c)). Both EMSA and yeast one-hybrid assays demonstrated that UvCGBP1 bound to the promoter regions of *UvPmk1* and *UvSlt2* (Figure S4(d,e,f)). Moreover, we also examined expression profiles of *UvPmk1* and *UvSlt2* during infection at 1–3 dpi in *∆UvCGBP1-33* and found expression of both these two genes at 3 dpi was significantly reduced in the ∆*UvCGBP1* mutant (Figure 7(a)). Overall, these results suggest that UvCGBP1 participates in the regulation of multiple MAPK pathways on the transcriptional level in *U. virens*.

### ∆UvPmk1 and ∆UvSlt2 completely lost virulence

To explore the role of *UvPmk1 and UvSlt2* in *U. virens* infection, we then conducted pathogenicity and plant infection assays of *∆UvPmk1-28* and *∆UvSlt2-2* mutants on rice susceptible cv. Wanxian-98. After 21 dpi, the WT produced approximately 50 false smut balls per panicle, whereas the *∆UvPmk1-28* and *∆UvSlt2-2* mutants failed to produce smut balls and were nonpathogenic (Figure 7(b)). For all tested strains, many hyphae were observed to extend on the surface of spikelets under SEM at 1 dpi. At 6 dpi, hyphae of the WT were found on the filaments but no hyphal could enter into the inner space of rice spikelets for the *∆UvPmk1-28* and *∆UvSlt2-2* mutants (Figure 7(c)). These results indicate that *UvPmk1* and *UvSlt2* are both essential for virulence of *U. virens.*

### Deletion of UvCGBP1 leads to reduced protein levels of p42/44 MAPK

To explore whether the knockout of *UvCGBP1* affected the protein levels of UvSlt2 and UvPmk1, we assayed with the anti-p42/44 MAPK specific antibody. The western blotting showed two bands, a 46 kDa one representing the protein level of UvSlt2, and a 42 kDa one representing the UvPmk1 protein level. The results revealed that protein level of UvPmk1 was decimated in the ∆*UvCGBP1-33* mutant, and UvSlt2 also showed a slight reduction (Figure 7(d)). These results suggested that the deletion of *UvCGBP1* reduced the protein levels of p42/44 MAPK.

### Overexpression of UvPmk1 in the *∆*UvCGBP1 partially restores impaired virulence

To further determine whether UvCGBP1 regulates the MAPK pathway to affect the pathogenicity of *U. virens*, overexpression of *UvPmk1* or *UvSlt2* was conducted in ∆*UvCGBP1-33* mutant (Figure S5(a,b)). We found that the growth rate of *OEUvPmk1* strains was partially restored and significantly higher than that of the ∆*UvCGBP1-33* mutant (Figure 7(e,f)). Importantly, compared with the ∆*UvCGBP1-33, OEUvPmk1* strains could form rice smut balls on all inoculated rice panicles and some false smut balls were found in diseased panicles (Figure 7(g,h,i)). The overexpression of *UvPmk1* in the ∆*UvCGBP1* restored partial virulence. These results further confirmed that UvCGBP1 regulated the MAPK pathway to affect the pathogenicity of *U. virens.*

## Discussion

In this study, we have identified a cutinase G-box binding protein, UvCGBP1 of which C_2_H_2_ zinc-finger domain is highly homologous to those of the fungal transcription factors Msn2p, Msn4p, MHY1p and Seb1p, but does not show any such similarity in other regions. The CGBP1 homologues are well conserved in other filamentous ascomycetes, such as *Neurospora crassa, Fusarium graminearum* and *M. oryzae*. However, none of these CGBP1 homologues in fungi have been functionally characterized. Here, we provide a comprehensive genome-wide binding map of UvCGBP1 and its regulatory network, and further describe a previously unknown regulatory role where UvCGBP1 mediates the MAPK pathway to regulate fungal virulence. As found previously, there are also a limited number of upstream transcription factors for MAPK signaling regulation in fungi. Our findings reveal insights into the mechanism of UvCGBP1-mediated gene regulation in fungal development and virulence.

To adapt to environmental changes, fungal pathogens should continually adjust their physiology to enhance infection and survival. At the molecular level, fungi possess complex signal transduction pathways that allow them to respond appropriately to alterations in external stimuli, including biotic and abiotic stresses, such as changes in oxidative stress and osmotic stress [42]. Reactive oxygen species (ROS) are well known to be important for various cellular functions in signaling and host defense [43]. When *U. virens* infects rice spikelets, a ROS burst might be triggered to resist pathogen invasion. After treatment with H_2_O_2,_ expression of *UvCGBP1* was strongly induced. We speculate that the ROS burst triggered in the rice host defense response activated the expression of this transcription factor, *UvCGBP1*, to facilitate infection. Otherwise, the AGGGG motif is an osmolarity stress response-related element [27], and UvCGBP1 may be involved in regulation of the osmolarity stress response. As expected, the osmotic stress agent NaCl strongly induced *UvCGBP1* expression in this study, and UvCGBP1 might bind to the AGGGG motif to regulate the high osmotic stress response. We hypothesize that during the interaction between rice and *U. virens*, the host could alter environmental pressures to resist the infection by *U. virens*, and *U. virens* may use some substances from rice to activate the expression of virulence factor UvCGBP1 and invade the host successfully.

The plant cuticle is the first physical defensive barrier to resist pathogen invasion and environmental stresses [44]. However, most fungal pathogens of plants produce cutinases that hydrolyze host cutin and promote fungal penetration [45]. Multiple copies of cutinase genes might exist in fungal genomes and the role of each cutinase gene could be different [46]. In the hemibiotrophic fungus *M. oryzae*, there are 16 putative cutinase genes in the genome database. Deletion of a cutinase gene *Mocut1* did not affect the virulence of *M. oryzae* [47], whereas the *Mocut2* deletion mutants showed decreased virulence [48]. Cutinase gene deletion did not affect *Botrytis cinerea* infection on gerbera flowers or tomato fruits [49]. In *U. virens*, four cutinases were identified with a highly conserved motif (GYSQG). However, each of the single cutinase genes was not necessary for virulence of *U. virens*. In this biotrophic fungus, cutinase seems not to play the main role in pathogenesis for rice smut ball formation. Due to the low genetic efficiency of transformation and knockout, multi-knockout is currently impractical in *U. virens*. Alternatively, to avoid functional redundancy of the other cutinase genes in *U. virens* in single cutinase gene deletion, the enzymatic activity of cutinase was evaluated in an *UvCGBP1* deletion mutant. Consistently, deletion of *UvCGBP1* did not affect the activity of cutinase. Hence, cutinase might not be considered as the main target for the loss of pathogenicity of *UvCGBP1* deletion mutants.

Identifying DNA-binding sites of transcription factors are critical to understand the molecular mechanisms underlying pathogenesis. Using ChIP-seq, we identified 865 target genes whose promoters were strongly bound by UvCGBP1. Interestingly, we found that UvCGBP1 binds to its own promoter and self-regulates its own expression (Figure S6). Thus, activation of UvCGBP1 by host or environmental stress might be balance-controlled to provide fine-tuned regulation during infection. Among the target genes, over 90 transcription factors were directly regulated by Uv*CGBP1* in *U. virens*, including C_2_H_2_-, GATA-, C6-type zinc finger protein, bZIP transcription factor, fungal specific transcription factor and others (Table S5). Within these, previous studies reported that the fungus-specific transcription factor *UvPro1* (*Uv8b_4390*) plays a key role in mycelial vegetative growth, conidial production, stress response, and virulence of *U. virens* [11]. There is an extensive network of genes involved with UvCGBP1 that are directly or indirectly regulated through other transcription factors. Other target genes are involved in various pathways, including autophagy, cAMP signaling pathway, MAPK signaling pathway, Ras signaling pathway, calcium signaling pathway, and metabolism in general. This observation led us to hypothesize that the transcription factor UvCGBP1 governs a complex regulatory network.

In eukaryotic organisms, the MAPK signaling pathway mediates responses to physiological and environmental signals [50,51]. In filamentous fungal pathogens, the Pmk1 MAPK pathway and the Slt2 MAPK pathway play a conserved role in virulence, while the Hog1 MAPK pathway has a species-specific role in virulence [51]. In *U. virens*, deletion of *UvPmk1* and *UvSlt2* involved loss of pathogenicity, which was in line with the results in other phytopathogens including *M. oryzae, Ustilago maydis, Colletotrichum gloeosporioides, B. cinerea* and *F. graminearum* [51]. In the *∆UvCGBP1-33* mutant, the protein levels of Pmk1 and Slt2 and the expression during infection were significantly reduced, but only overexpression of *UvPmk1* in ∆*UvCGBP1-33* mutant could restore partial virulence. We speculate that it might be due to phosphorylation events of their downstream targets. In future studies, the phosphorylation of p44/42 MAP kinase downstream targets needs to be investigated.

Rice resistance and defense-regulator genes confer resistance to rice pathogens [52]. However, few cultivars and resistance genes have been applied for breeding rice cultivars with stable resistance to RFS. Therefore, it is critical to develop alternatives to control this disease. Host-induced gene silencing (HIGS) is an effective approach to generate resistant cultivar for fungal disease control, and key genes that function in *U. virens* development or pathogenesis could serve as targets for the control of fungal diseases via HIGS. Small interference RNAs in transgenic host plants could match pathogen important genes and silence fungal genes during infection [53,54]. For example, virulence genes *Chs3b, CYP51, SGE1, FGP1* and *STE12* as HIGS target confers wheat resistance to *Fusarium graminearum* [55–57]. Effector gene *Avra10* as an HIGS target in wheat affects the development of *Blumeria graminis* [58]. In the current study, deletion of *UvCGBP1* caused severe defects in development and virulence of *U. virens*. Importantly, this protein is ascomyce-specific and has not been found in any animals and plants. It led us to believe that the *UvCGBP1* could be an important possible RNAi target for reducing rice false smut development in rice.

In conclusion, we identified one potential mechanism by which the novel transcription regulator UvCGBP1 regulates development and virulence. UvCGBP1 mainly recognizes and binds the AGGGG cis-element, and it regulates itself and other target genes involved in various aspects of functions. However, UvCGBP1 might regulate virulence independent of cutinase genes. We identified the genome-wide targets of UvCGBP1, and found that it plays an important role as a transcriptional regulator in the response to numerous signaling pathways, such as the Pmk1 MAP kinase cascade. We confirmed that UvCGBP1 regulates virulence through mediating the Pmk1 MAPK pathway. To our knowledge, a limited transcription factors in upstream of the MAPK pathway was identified in fungi. Our study provides new insight into the mechanism of UvCGBP1-mediated gene regulation in fungal development and virulence.

## Supplementary Material

Supplemental Material
