# Supplementary figures and images for: A novel transcription factor UvCGBP1 regulates development and virulence of rice false smut fungus Ustilaginoidea virens
Source: Virulence. 2021 Aug 4;12(1):1563–79. doi: 10.1080/21505594.2021.1936768 (PMC8344781; doi:10.1080/21505594.2021.1936768)

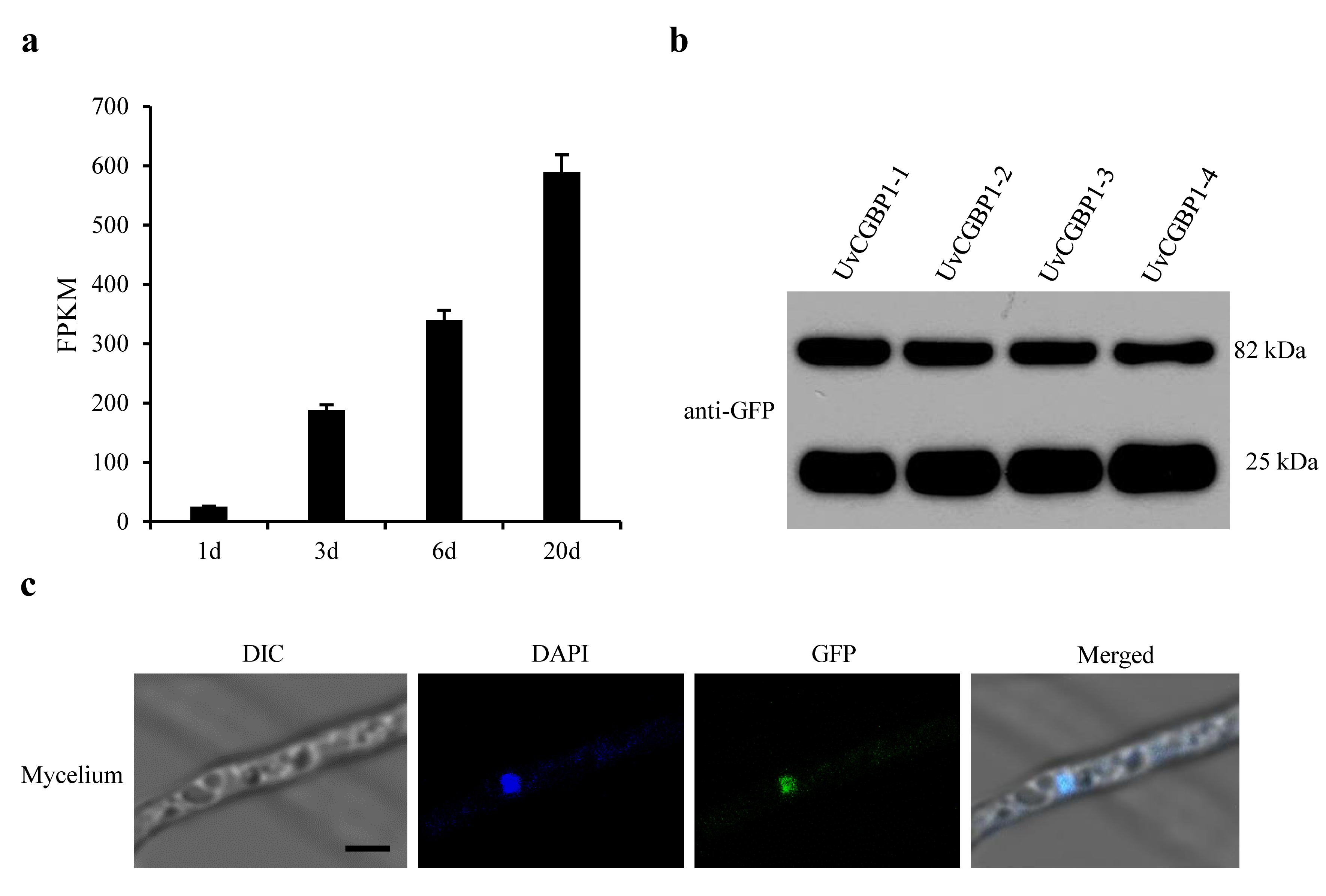

Supplement: Supplemental Material [file KVIR_A_1936768_SM2191.zip › supplementary/Figure S1.tif]

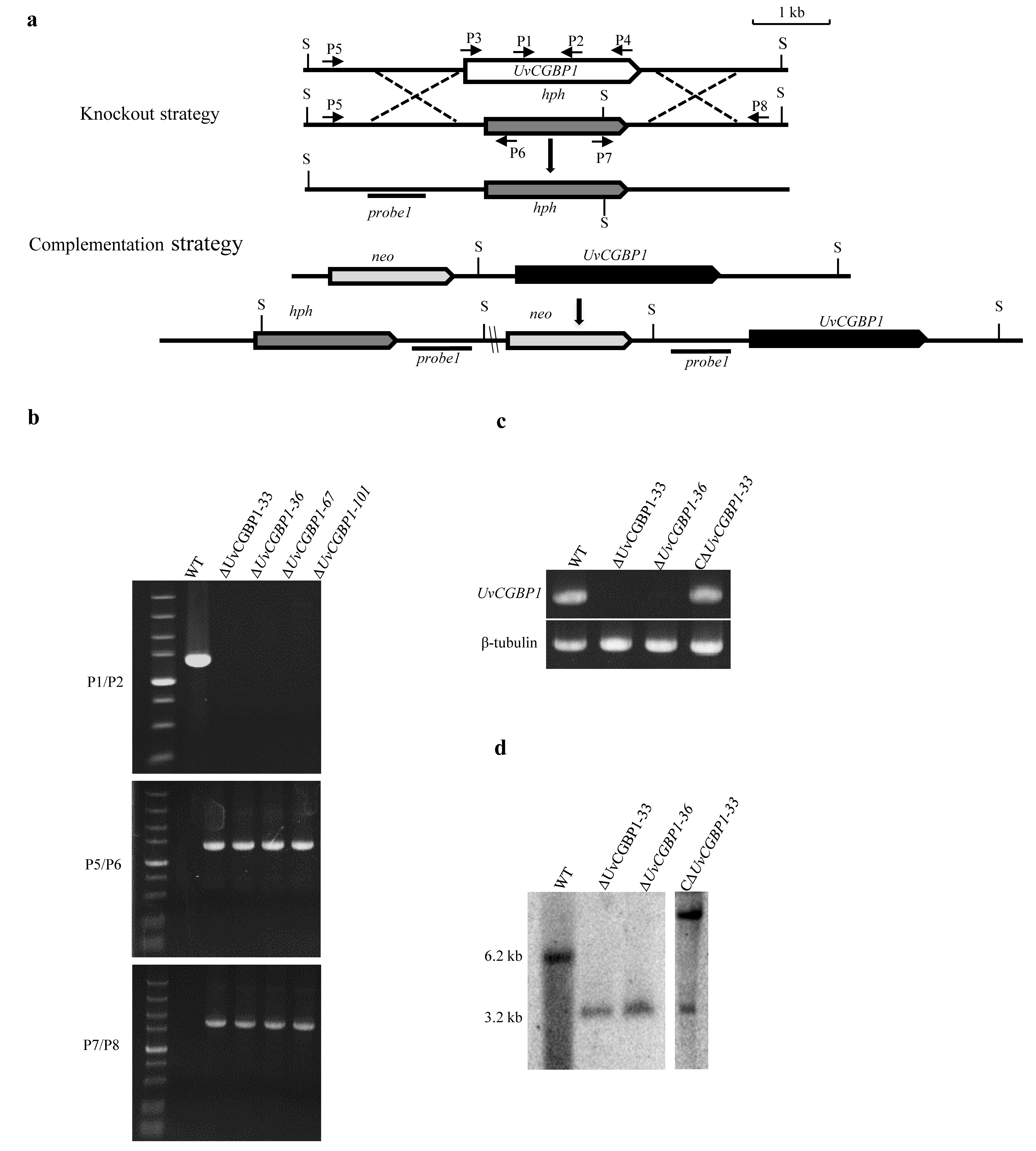

Supplement: Supplemental Material [file KVIR_A_1936768_SM2191.zip › supplementary/Figure S2.tif]

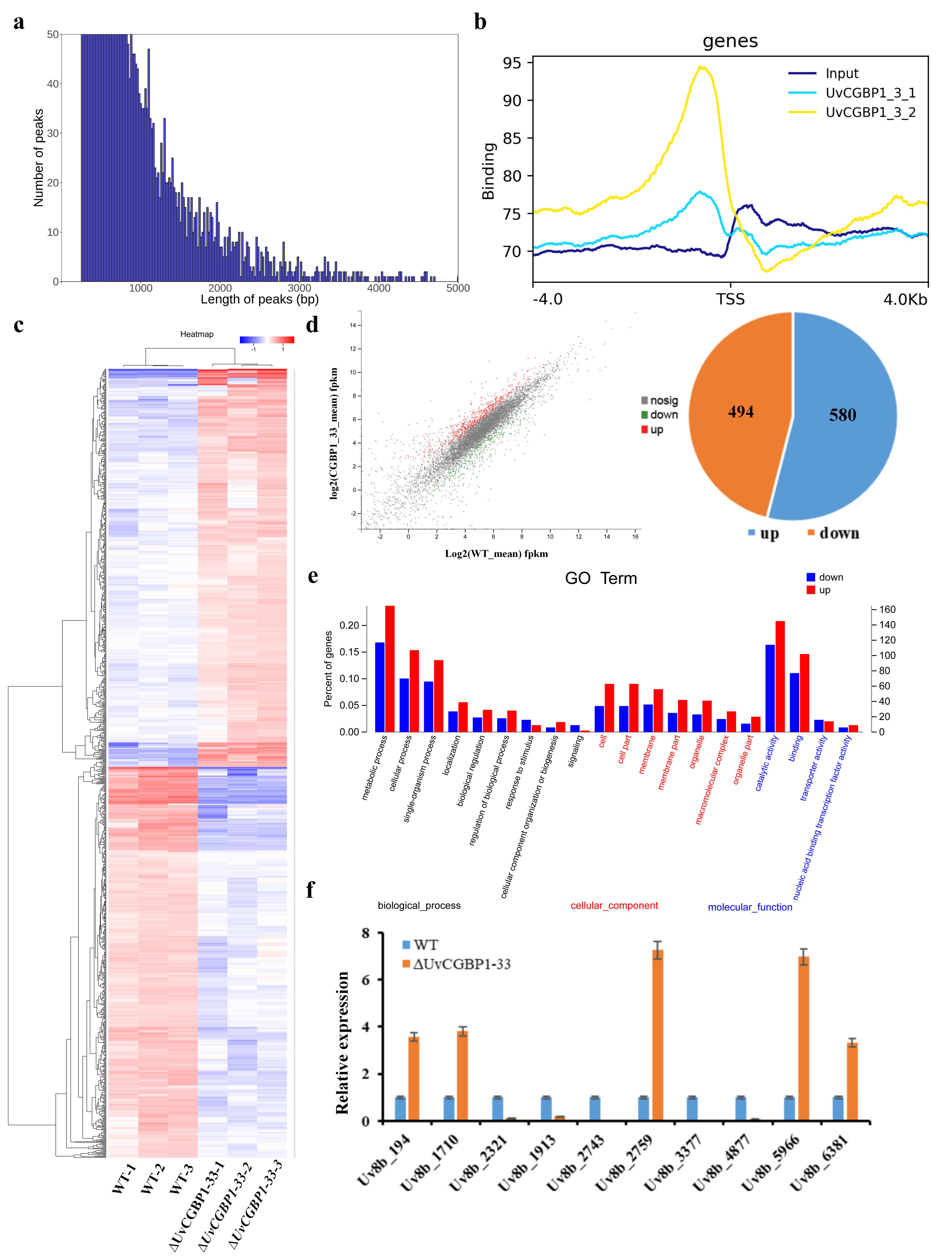

Supplement: Supplemental Material [file KVIR_A_1936768_SM2191.zip › supplementary/Figure S3.tif]

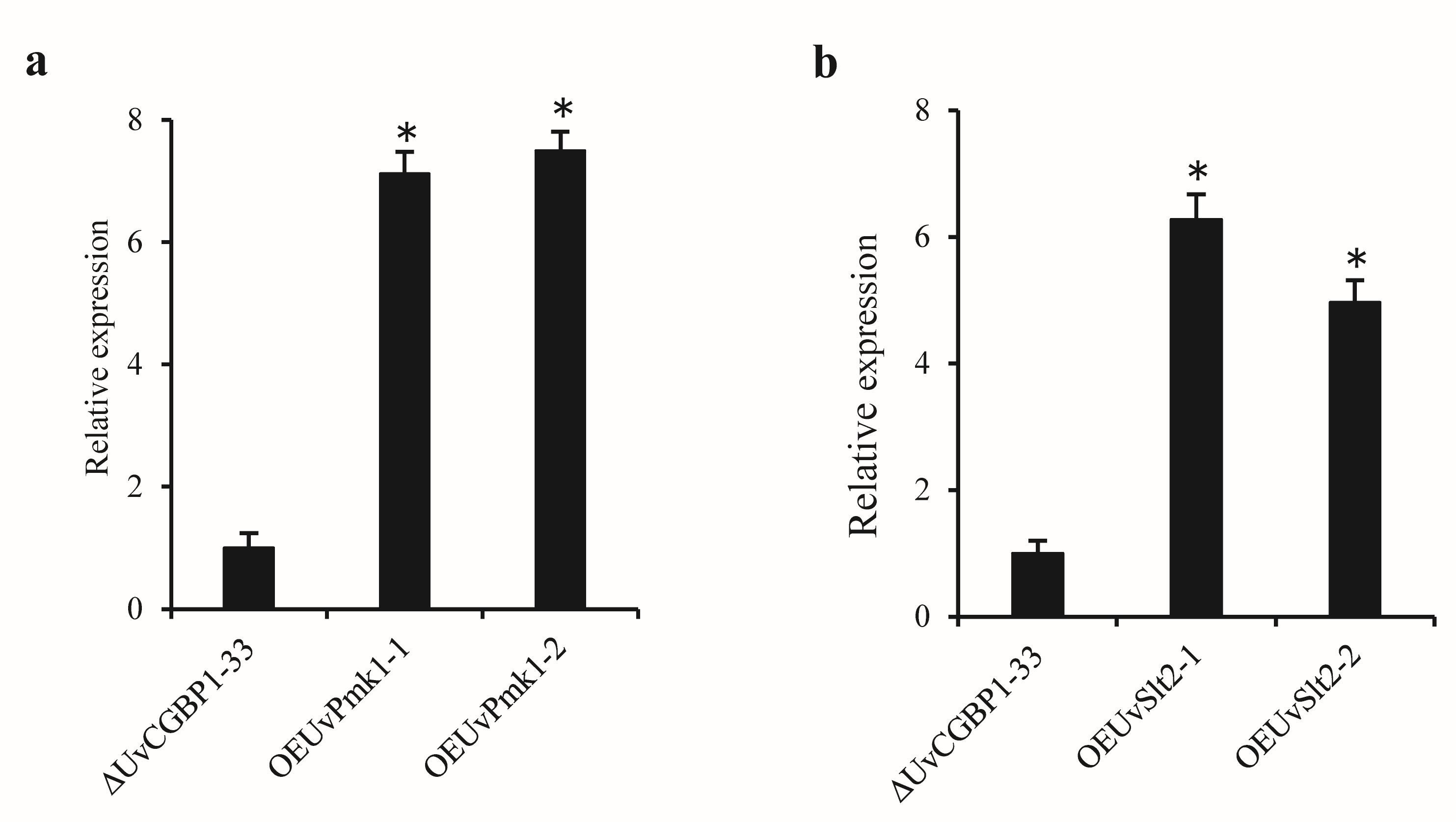

Supplement: Supplemental Material [file KVIR_A_1936768_SM2191.zip › supplementary/Figure S5.tif]

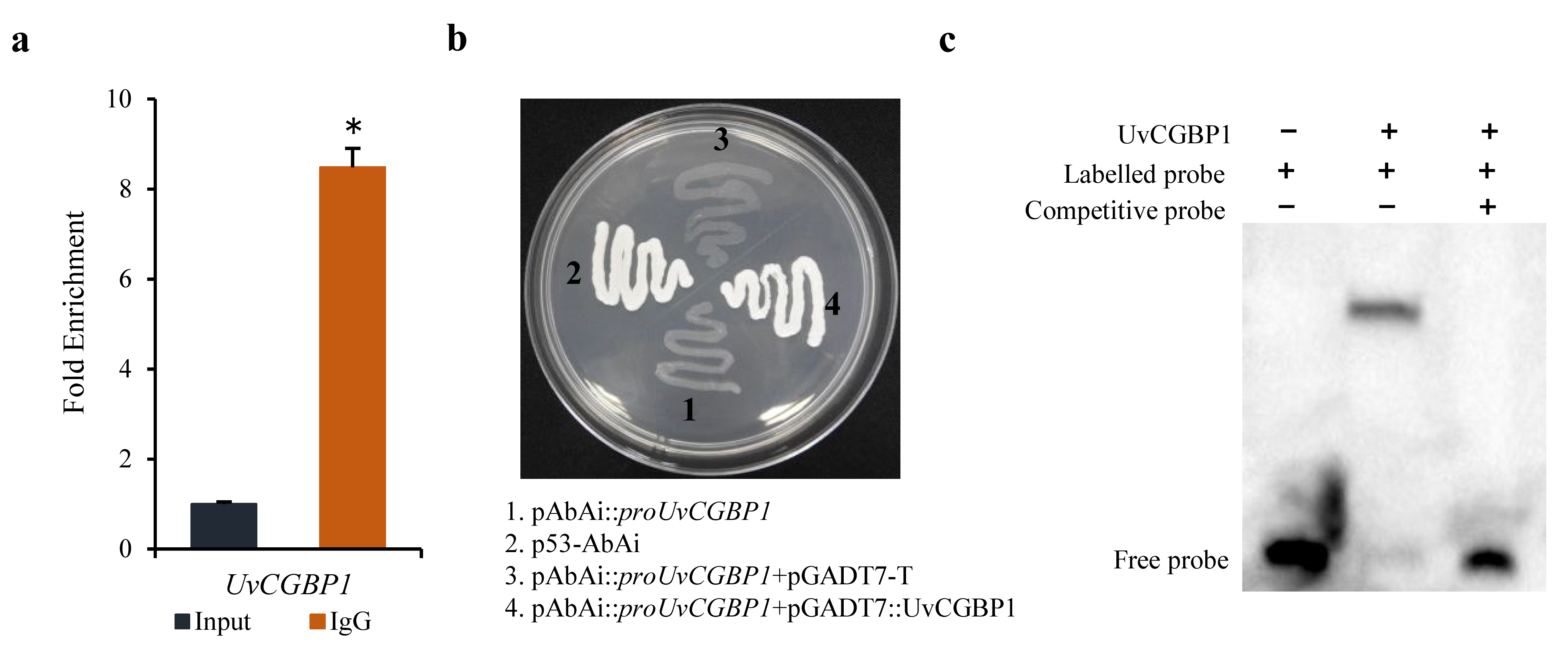

Supplement: Supplemental Material [file KVIR_A_1936768_SM2191.zip › supplementary/Figure S6.tif]
