# Supplementary material for: A novel transcription factor UvCGBP1 regulates development and virulence of rice false smut fungus Ustilaginoidea virens
Source: Virulence. 2021 Aug 4;12(1):1563–79. doi: 10.1080/21505594.2021.1936768 (PMC8344781; doi:10.1080/21505594.2021.1936768)
Supplement: Supplemental Material [file KVIR_A_1936768_SM2191.zip › supplementary/Table S1.doc]

| **Table S1.** Primers used in this study. | | | |
| --- | --- | --- | --- |
| **Primer** | **Sequence (5’-3’)** | **Primer** | **Sequence (5’-3’)** |
| F1F | GGGGTACCGTGGCTGTGCGATGACG | F1R | CCAAGCTTCGCGGATTGCTGCTTTA |
| F2F | CGGGATCCCTCGGCAAAGGTGGACTG | F2R | GCTCTAGAAGCCCTCTGTGCTTGTGGT |
| P1 | TTCTGTCGTTTCGCTGGGTC | P2 | CCGTAGTTGGCAAAGTCCTCC |
| P3 | ATGTCTCAAGCTGCTGCT | P4 | GTCGGAGCGCTTTCGCTT |
| P5 | CCAAGCTTTCTGCACGCCCACGTCA | P6 | AAATCGCCCGCAGAAGC |
| P7 | TGCCTAAATGAACCATCTTGTC | P8 | TGCAACTTGGCCTGGATT |
| Probe1 | GAACACGGAGATGCAGTTGA | Probe2 | GATAGGGTACGCCTTGGTAGA |
| ^a^5800F | TTCTGTCGTTTCGCTGGGTC | ^a^5800R | GCTTATCCTGGTGCGTGTCC |
| ^a^2652F | AAAGGCTGACATGAGTTCCCT | ^a^2652R | CTCCTTCACGCGAGGTCCGTA |
| ^a^7184F | TTATCCGGCCTCCTTCTCCGAT | ^a^7184R | TGCAAATCCCTTTTCGTCCGAG |
| ^a^7527F | TCGTCCTGTTCGGATACACCA | ^a^7527R | CAAACCATGTCGCCGGTGTT |
| ^a^8131F | TTCCGTCTTTTCCCCTTGGTC | ^a^8131R | CGCGGCTTTCTCATACCGTTG |
| ^a^194F | CTGCGGGACGATCACTCTT | ^a^194R | CACTTGGTCGGGATTGGGT |
| ^a^1710F | CTGCTGCGTCCGTTACTTCG | ^a^1710R | GCATAGCCGTTGCTCTTGG |
| ^a^2321F | CTCACGATGCCGTCACCA | ^a^2321R | CCGAGGCGTAGTTGGGTA |
| ^a^1913F | TTTACAAAGGGTTCGTTGGTCC | ^a^1913R | CCGGCTTCATCAGGTCTGC |
| ^a^2743F | ATGCCGCCATTCTCCCTC | ^a^2743R | TCCCGAGCAGCCTAAACC |
| ^a^2759F | CTGGCTTTCTCGGTGGTTAT | ^a^2759R | GATGATGCTGCTTCGTGTTTT |
| ^a^3377F | AAGGATGCCCTGCTACCG | ^a^3377R | TGACCGCCCAAGTTTCG |
| ^a^4877F | ACCTCAACGGACACCTCG | ^a^4877R | GCACCTTCTTCCCTCTGC |
| ^a^5966F | CGCTTCAAGGGTATCCGTGTC | ^a^5966R | GGCGAGCCATTGGCAGTT |
| ^a^6381F | GTCTAAGGGAGCGTCCGAGTC | ^a^6381R | TGTCCTGCCGTTGGGTGA |
| ^a^4095F | AAAGGAGCTGGGACAGGGTG | ^a^4095R | GGTGACTTTCTTGATGGCAACG |
| ^b^2494LF | AATAAGGGCAGGGGATAAAGTCGGGGGGGGGTAAAAAG | ^b^2494LR | CTTTTTACCCCCCCCCGACTTTATCCCCTGCCCTTATT |
| ^b^4095LF | GGCCGGGTGAGACGGCCAGGCCGGGGGACCTGGTGATTG | ^b^4095LR | CAATCACCAGGTCCCCCGGCCTGGCCGTCTCACCCGGCC |
| ^b^1729LF | AAGTCTAGTGGAGTGTTGGACCCCCCCCTTTCCTCTCATCTGACA | ^b^1729LR | TGTCAGATGAGAGGAAAGGGGGGGGTCCAACACTCCACTAGACTT |
| b7791LF | GTTCCTGGACGTTGTCTGGGGTTGGGGGGAAGGGGGGTTGTGGCAGCCGA | b7791LR | TCGGCTGCCACAACCCCCCTTCCCCCCAAC CCCAGACAACGTCCAGGAAC |
| ^b^Pro1LF | GATTCACACAGTCTGCCCCCCCCTTGCCCCCCCCCCCCCCCAAAACCGTTCAC | ^b^Pro1LR | GTGAACGGTTTTGGGGGGGGGGGGGGGCAAGGGGGGGGCAGACTGTGTGAATC |
| ^b^Pal1LF | GTTGCGCTTAACGGGGTTTCCCCCCCTCCTCACACCGCACCGCACCGCAC | ^b^Pal1LR | GTGCGGTGCGGTGCGGTGTGAGGAGGGGGGGAAACCCCGTTAAGCGCAAC |
| ^b^Cdc11LF | TGCACCTCCTCCCGCCCCCTTTGCCCTGGCCCAGCTTGTCCAAGA | ^b^Cdc11LR | TCTTGGACAAGCTGGGCCAGGGCAAAGGGGGCGGGAGGAGGTGCA |
| ^a^ Primers of qRT-PCR were used to detect the expression level of related genes. | | | |
| ^b^ The 5'-ends of these primers were modified with biotin for EMSA detection probes. | | | |
