# Supplementary material for: A novel transcription factor UvCGBP1 regulates development and virulence of rice false smut fungus Ustilaginoidea virens
Source: Virulence. 2021 Aug 4;12(1):1563–79. doi: 10.1080/21505594.2021.1936768 (PMC8344781; doi:10.1080/21505594.2021.1936768)
Supplement: Supplemental Material [file KVIR_A_1936768_SM2191.zip › supplementary/Table S2.doc]

| Table S2. ChIP-seq data statistics of the UvCGBP1. | | | | | | | | |
| --- | --- | --- | --- | --- | --- | --- | --- | --- |
| **Sample** | **Total reads** | **Mapped reads** | **Percent of mapped reads** | **Properly paired** | **Singletons** | **With itself and mate mapped paired reads** | **With mate mapped to different chr paired reads** | **With mate mapped to different chr quality paired reads** |
| Input | 49,020,998 | 41,379,618 | 84.41 | 38,500,146 | 1,384,332 | 39,876,292 | 1,069,578 | 722,033 |
| UvCGBP1_3_1 | 47,509,303 | 36,955,880 | 77.79 | 34,770,480 | 1,189,787 | 35,680,610 | 705,572 | 452,970 |
| UvCGBP1_3_2 | 43,601,599 | 26,741,675 | 61.33 | 25,052,452 | 998,614 | 25,680,948 | 482,736 | 297,853 |
