# Supplementary material for: A novel transcription factor UvCGBP1 regulates development and virulence of rice false smut fungus Ustilaginoidea virens
Source: Virulence. 2021 Aug 4;12(1):1563–79. doi: 10.1080/21505594.2021.1936768 (PMC8344781; doi:10.1080/21505594.2021.1936768)
Supplement: Supplemental Material [file KVIR_A_1936768_SM2191.zip › supplementary/Table S3.doc]

| Table S3. RNA sequencing data statistics of the WT and ΔUvCGBP1-33 mutant. | | | | | | | | |
| --- | --- | --- | --- | --- | --- | --- | --- | --- |
| **Sample** | **Raw reads** | **Raw bases** | **Clean reads** | **Clean bases** | **Error rate (%)** | **Q20 (%)** | **Q30 (%)** | **GC content (%)** |
| CGBP1_33_1 | 58,032,440 | 8,762,898,440 | 57,239,460 | 8,515,516,794 | 0.0241 | 98.36 | 95.07 | 56.24 |
| CGBP1_33_2 | 58,714,542 | 8,865,895,842 | 57,995,554 | 8,641,815,278 | 0.0239 | 98.44 | 95.25 | 56.84 |
| CGBP1_33_3 | 51,056,502 | 7,709,531,802 | 50,344,186 | 7,486,946,881 | 0.0242 | 98.35 | 94.96 | 57.1 |
| WT_1 | 55,498,728 | 8,380,307,928 | 54,822,442 | 8,153,746,839 | 0.0241 | 98.37 | 95.06 | 56.5 |
| WT_2 | 59,192,008 | 8,937,993,208 | 58,459,516 | 8,727,609,749 | 0.0252 | 97.97 | 93.91 | 56.14 |
| WT_3 | 52,044,626 | 7,858,738,526 | 51,390,426 | 7,665,407,098 | 0.0251 | 98 | 93.98 | 55.7 |
