# Supplementary material for: A novel transcription factor UvCGBP1 regulates development and virulence of rice false smut fungus Ustilaginoidea virens
Source: Virulence. 2021 Aug 4;12(1):1563–79. doi: 10.1080/21505594.2021.1936768 (PMC8344781; doi:10.1080/21505594.2021.1936768)
Supplement: Supplemental Material [file KVIR_A_1936768_SM2191.zip › supplementary/Table S4.doc]

| Table S4. UvCGBP1 regulation target genes from MAPK pathway. | | | |
| --- | --- | --- | --- |
| **Gene ID** | **Flanking gene description** | **Peak location** | **ChIP-seq fold enrichment** |
| Uv8b_197 | Cytokinesis protein | JHTR01000001.1: 641,641-643,207 | 3.43433 |
| Uv8b_447 | Putative protein-tyrosine-phosphatase | JHTR01000001.1: 1,936,273-1,937,801 | 4.70193 |
| Uv8b_1111 | Putative two-component response regulator SSK1p | JHTR01000003.1: 871,872-872,265 | 2.02195 |
| Uv8b_1255 | Putative Rho GTPase activator (Sac7) | JHTR01000004.1: 384,634-385,190 | 2.20047 |
| Uv8b_1325 | MAP kinase kinase kinase Ste11 | JHTR01000004.1: 599,171-601,958 | 3.40725 |
| Uv8b_1796 | MADS box protein | JHTR01000007.1: 185,140-186,443 | 2.61978 |
| Uv8b_1842 | MAP kinase activator | JHTR01000007.1: 334,228-335,675 | 2.68987 |
| Uv8b_1888 | Stress-activated MAP kinase | JHTR01000007.1: 509,790-512,017 | 2.877129 |
| Uv8b_1901 | Regulator of G protein signalling pathway | JHTR01000007.1: 555,172-556,138 | 7.06182 |
| Uv8b_2080 | MADS-box MEF2 type transcription factor | JHTR01000008.1: 291,117-292,519 | 3.4427 |
| Uv8b_2494 | Mitogen activated protein kinase | JHTR01000009.1: 832,473-833,353 | 3.10307 |
| Uv8b_2516 | MAPKKK cascade protein kinase regulator Ste50 | JHTR01000010.1: 24,496 -25,257 | 2.79631 |
| Uv8b_2520 | G protein beta subunit | JHTR01000010.1: 35,203 -36,714 | 3.00378 |
| Uv8b_2688 | Putative protein-tyrosine phosphatase | JHTR01000011.1: 26,709 -27,305 | 1.8676 |
| Uv8b_2759 | Glycerol-3-phosphate dehydrogenase | JHTR01000011.1: 222,657-223,491 | 8.57778 |
| Uv8b_3608 | RhoGAP domain containing protein | JHTR01000015.1: 323,336-326,265 | 4.84746 |
| Uv8b_4095 | Putative MAP kinase | JHTR01000019.1: 112,415-113,495 | 10.4966 |
| Uv8b_428 | Cell division control protein | JHTR01000001.1: 1,748,370-1,749,357 | 3.29789 |
| Uv8b_4772 | 14-3-3-like protein | JHTR01000023.1:487,405-487,700 | 1.63612 |
| Uv8b_5009 | Phosphatidylinositol 3-kinase 3 | JHTR01000026.1: 62,537 -63,089 | 2.24637 |
| Uv8b_5857 | Protein kinase C | JHTR01000033.1: 151,060-151,718 | 1.36005 |
| Uv8b_6200 | Putative histidine phosphotransferase HPT1p | JHTR01000037.1: 200,660-202,665 | 6.00129 |
| Uv8b_6310 | Cell division control protein | JHTR01000039.1: 69,182 -69,649 | 2.45093 |
| Uv8b_6524 | Ubiquitin ligase (Nedd4/Rsp5p) | JHTR01000041.1: 220,401-221,556 | 2.78573 |
| Uv8b_6810 | MAP kinase kinase kinase Czk3 | JHTR01000046.1: 77,522 -78,469 | 1.93674 |
| Uv8b_7010 | Putative protein kinase | HTR01000049.1: 172,339-175,774 | 5.9249 |
| Uv8b_7103 | Putative histidine kinase HHK5p | JHTR01000051.1: 203,346-204,020 | 1.89956 |
| Uv8b_7405 | Transcriptional repressor tup1 | JHTR01000061.1: 1,941-3,245 | 2.98588 |
| Uv8b_7445 | G protein gamma subunit | JHTR01000061.1: 131,384-133,000 | 3.92901 |
| Uv8b_7728 | Transcription factor Fst12 | JHTR01000071.1: 70,866 -72,988 | 3.21427 |
| Uv8b_7744 | Hypothetical protein | JHTR01000071.1: 121,806-122,329 | 2.21124 |
| Uv8b_7754 | Catalase | JHTR01000071.1: 144,086-146,797 | 3.86675 |
| Uv8b_7960 | Putative OPY2 protein | JHTR01000077.1: 126,870-128,081 | 3.01596 |
| Uv8b_8233 | Putative casein kinase | JHTR01000092.1: 72,023-73,335 | 3.09052 |
| Uv8b_860 | Ste20-like protein | JHTR01000003.1: 57,853-61,095 | 2.98418 |
