# Supplementary material for: A novel transcription factor UvCGBP1 regulates development and virulence of rice false smut fungus Ustilaginoidea virens
Source: Virulence. 2021 Aug 4;12(1):1563–79. doi: 10.1080/21505594.2021.1936768 (PMC8344781; doi:10.1080/21505594.2021.1936768)
Supplement: Supplemental Material [file KVIR_A_1936768_SM2191.zip › supplementary/Table S5.doc]

| Table S5. ChIP-seq identified transcription factors as regulation targets by UvCGBP1. | | |
| --- | --- | --- |
| **Gene ID** | **Flanking gene description** | **ChIP-seq fold enrichment** |
| Uv8b_112 | N-terminal fungal transcription regulatory domain-containing protein | 2.34902 |
| Uv8b_119 | Fungal transcriptional regulatory protein | 1.52136 |
| Uv8b_135 | PHD transcription factor | 2.48509 |
| Uv8b_314 | bZIP transcription factor | 4.28828 |
| Uv8b_332 | Pre-mRNA splicing factor CEF1 | 2.71441 |
| Uv8b_336 | Acriflavine sensitivity control protein acr-2 | 2.12558 |
| Uv8b_462 | Transcription factor | 3.55 |
| Uv8b_466 | Putative Zn cluster transcription factor Rds2 | 1.57246 |
| Uv8b_492 | Mitochondrion organization and biogenesis protein | 3.295 |
| Uv8b_717 | Putative ubiquitin-protein ligase (Hul4) | 2.01796 |
| Uv8b_750 | Putative Myb-like DNA-binding protein | 1.6877 |
| Uv8b_836 | Putative Fungal specific transcription factor | 1.58876 |
| Uv8b_892 | Transcription factor TamA | 5.01357 |
| Uv8b_918 | Transcription elongation factor s-ii | 1.53828 |
| Uv8b_928 | Putative jumonji domain containing 2D | 4.36613 |
| Uv8b_1050 | Putative C6 transcription factor | 2.37111 |
| Uv8b_1060 | Aspartyl-tRNA synthetase | 1.83164 |
| Uv8b_1075 | Transcriptional activator hac1 | 8.47328 |
| Uv8b_1101 | Putative C6 transcription factor | 1.57111 |
| Uv8b_1287 | MYB and HSA domain protein | 1.76371 |
| Uv8b_1298 | Zinc finger transcription factor 1 | 2.88465 |
| Uv8b_1304 | DNA polymerase epsilon subunit C | 2.92364 |
| Uv8b_1333 | Homeodomain-containing protein | 2.36889 |
| Uv8b_1435 | Putative C2H2 finger domain protein | 4.72866 |
| Uv8b_1493 | C6 transcription factor | 2.85764 |
| Uv8b_1557 | Putative C2H2 finger domain protein | 2.77958 |
| Uv8b_1609 | Putative negative acting factor | 2.50548 |
| Uv8b_1784 | bZIP transcription factor (Fcr3) | 2.97709 |
| Uv8b_1794 | Putative C2H2 finger domain protein | 5.13763 |
| Uv8b_1796 | MADS box protein | 2.61978 |
| Uv8b_1901 | Regulator of G protein signalling pathway | 7.06182 |
| Uv8b_1944 | C6 zinc finger domain protein | 2.92632 |
| Uv8b_2080 | MADS-box MEF2 type transcription factor | 3.4427 |
| Uv8b_2167 | Positive regulator of purine utilization | 2.01577 |
| Uv8b_2192 | Putative C2H2 transcription factor (Egr2) | 3.27654 |
| Uv8b_2201 | bHLH family transcription factor | 6.02417 |
| Uv8b_2421 | N-terminal binuclear Zn cluster-containing protein | 4.13288 |
| Uv8b_2579 | Palmitoyltransferase pfa4 | 2.4218 |
| Uv8b_2671 | Cutinase transcription factor 1 beta | 12.70256 |
| Uv8b_2987 | C2H2 type zinc finger domain-containing protein | 5.81607 |
| Uv8b_3064 | Glutamate-cysteine ligase catalytic subunit | 4.33489 |
| Uv8b_3133 | Putative C6 transcription factor | 2.045159 |
| Uv8b_3206 | Ctf1 transcription factor | 2.62686 |
| Uv8b_3245 | Transcriptional activator xlnR | 2.5701 |
| Uv8b_3411 | SWIRM domain-containing protein | 3.86759 |
| Uv8b_3689 | C2H2 transcription factor | 2.70522 |
| Uv8b_3779 | Cross-pathway control 1 | 4.12983 |
| Uv8b_3807 | Helix-loop-helix DNA-binding domain-containing protein | 3.50974 |
| Uv8b_3851 | Putative Fungal specific transcription factor | 2.72078 |
| Uv8b_3866 | Putative signal transducer | 2.43762 |
| Uv8b_4006 | Palmitoyltransferase PFA3 | 5.2116 |
| Uv8b_4140 | Multiprotein-bridging factor 1 | 2.23841 |
| Uv8b_4383 | CCAAT-binding protein subunit HAP3 | 2.5649 |
| Uv8b_4390 | Transcriptional regulatory protein pro-1 | 2.35617 |
| Uv8b_4419 | CCAAT-binding complex subunit HAP2 | 2.87273 |
| Uv8b_4500 | Flocculation suppression protein | 4.87421 |
| Uv8b_4608 | Putative UV excision repair protein (RadW) | 2.67385 |
| Uv8b_4883 | Fungal specific transcription factor domain-containing protein | 6.75825 |
| Uv8b_4997 | GTPase-activating protein GYP5 | 2.1172 |
| Uv8b_5021 | C6 finger domain protein | 2.39894 |
| Uv8b_5144 | Zinc finger protein | 2.47317 |
| Uv8b_5298 | Zinc knuckle domain containing protein | 3.1487 |
| Uv8b_5300 | C6 zinc finger domain containing protein | 3.04496 |
| Uv8b_5421 | ARID/BRIGHT DNA binding domain containing protein | 2.75353 |
| Uv8b_5496 | Zinc finger protein | 2.24551 |
| Uv8b_5506 | C2H2 type zinc finger containing protein | 3.52193 |
| Uv8b_5567 | Putative homeobox transcription factor | 2.38777 |
| Uv8b_5734 | Putative bZip transcription factor | 2.04871 |
| Uv8b_5761 | Putative PHD finger and BAH domain protein (Snt2) | 2.3388 |
| Uv8b_5800 | Cutinase G-box binding protein | 1.39045 |
| Uv8b_5849 | Nitrogen response regulator | 2.00429 |
| Uv8b_5932 | C2H2 type zinc finger containing protein | 3.40731 |
| Uv8b_5933 | Siderophore regulation protein | 4.26703 |
| Uv8b_6009 | Fungal specific transcription factor domain protein | 2.94318 |
| Uv8b_6099 | Putative finger protein AZF1 | 3.44118 |
| Uv8b_6214 | Transcription factor, MYB family | 2.88591 |
| Uv8b_6591 | DNA binding regulatory protein AmdX | 2.37461 |
| Uv8b_6675 | Putative transcription factor | 3.14419 |
| Uv8b_6694 | Putative Cutinase transcription factor 1 alpha | 2.85066 |
| Uv8b_6741 | mRNA splicing factor RNA helicase | 3.42548 |
| Uv8b_6830 | Nucleosome binding protein | 2.71075 |
| Uv8b_6835 | Negative acting factor | 2.39911 |
| Uv8b_7027 | Fungal transcriptional regulatory protein | 5.17003 |
| Uv8b_7144 | Rho-GTPase-activating protein 8 | 5.72194 |
| Uv8b_7146 | 30S ribosomal protein S13 | 4.75234 |
| Uv8b_7289 | Multisynthetase complex auxiliary component p43 | 2.10629 |
| Uv8b_7324 | Carbon response regulator | 4.63601 |
| Uv8b_7403 | Transcriptional activator hac1 | 5.81039 |
| Uv8b_7573 | Fungal Zn binuclear cluster domain containing protein | 3.89804 |
| Uv8b_7718 | ssDNA binding protein | 2.24143 |
| Uv8b_7728 | Transcription factor Fst12 | 3.21427 |
| Uv8b_7979 | Fungal transcriptional regulatory protein | 5.42121 |
